# Validation of a CD81-based flow cytometry assay to assess dCas9 silencing activity

**DOI:** 10.1186/s13104-026-07796-5

**Published:** 2026-04-08

**Authors:** Iris Manosalva, Megan Charles-Alfred, Magali Torres, Joaquin Felipe Roca Paixao, Salvatore Spicuglia

**Affiliations:** 1 Inserm, Theories and Approaches of Genomic Complexity (TAGC), UMR1090, Aix-Marseille University, 13288 Marseille, France; 2 Equipe Labellisée LIGUE Contre le Cancer, 13288 Marseille, France

**Keywords:** CRISPRi, CD81, Silencing

## Abstract

**Objective:**

The efficiency of CRISPR interference (CRISPRi) depends on functional dCas9 activity, yet practical and reproducible validation of dCas9-expressing cell lines remains limited. Here, we describe a simple and reproducible assay to assess dCas9 functionality using a single sgRNA targeting the nonessential and ubiquitously expressed surface protein CD81.

**Results:**

We evaluated this approach in multiple hematological and solid tumor cell lines expressing the dCas9-KRAB-MeCP2 repressor complex. In all tested models, CD81 targeting resulted in a consistent reduction of surface protein levels, quantified by flow cytometry. This assay provides a rapid and quantitative functional readout of dCas9 activity without the need for reporter constructs or transcriptional assays. The CD81-targeting sgRNA and validated cell lines are made available to support reproducibility and technical standardization in CRISPRi experiments. This strategy can be readily implemented in any laboratory using CRISPRi-based approaches.

**Supplementary Information:**

The online version contains supplementary material available at 10.1186/s13104-026-07796-5.

## Introduction

For many years, numerous techniques have been developed to better understand the mechanisms of gene function. One of the most important discoveries in this area has been the CRISPR/Cas9 system, which allows for efficient genome editing. With this method, it is possible to replace a portion of the genome with another sequence or induce loss of gene activity by creating a double-strand break, followed by homologous recombination that disrupts gene function [1, 2]. However, despite its efficiency, the CRISPR/Cas9 system also has certain limitations, such as the potential for off-target effects due to unintended cuts at non-specific genomic locations [3]. To overcome this, the dead Cas9 (dCas9) system was developed [4]. This version of mutated Cas9 lacks nuclease activity and does not cut DNA, but it can still bind to specific genomic sequences. When fused to transcriptional repressor domains (CRISPRi), like KRAB or MeCP2, dCas9 can effectively reduce gene expression [5, 6]. A main advantage of the CRISPRi system is that it can target both coding and non-coding regions of the genome, expanding its potential applications in functional genomics and epigenetic regulation [7, 8]. This represents a powerful technological tool for experimental biology.

One of the challenges in developing CRISPR/Cas9 systems is to obtain efficient Cas9 or dCas9 activity [7]. A common strategy to assess CRISPRi efficiency is to double transfect cells with a vector expressing the GFP marker and a sgRNA targeting the promoter of the GFP vector [6]. Alternative approaches include transcriptional assays such as RT-qPCR, which measure the reduction of target mRNA levels. These strategies can be indirect, time-consuming, or dependent on artificial constructs that may not fully reflect endogenous gene regulation. An alternative is to assess the expression of a ubiquitously expressed endogenous gene. CD81 is a non-essential membrane protein that is broadly expressed and can be easily detected by flow cytometry [9]. CRISPR-mediated inactivation of *CD81* has been previously used to evaluate CRISPRi efficiency [9–11]. Here, we confirmed the use of a single guide RNA (sgRNA) targeting the human *CD81* promoter to assess CRISPRi efficiency in several clonal cell lines expressing dCas9-KRAB-MeCP2 (dCas9-KM) cassettes. This approach makes CD81 an ideal marker for evaluating dCas9-mediated repression in live cells across multiple cellular contexts.

## Results and discussion

### Targeting CD81 as a universal marker for dCas9 efficiency

**Fig. 1:**
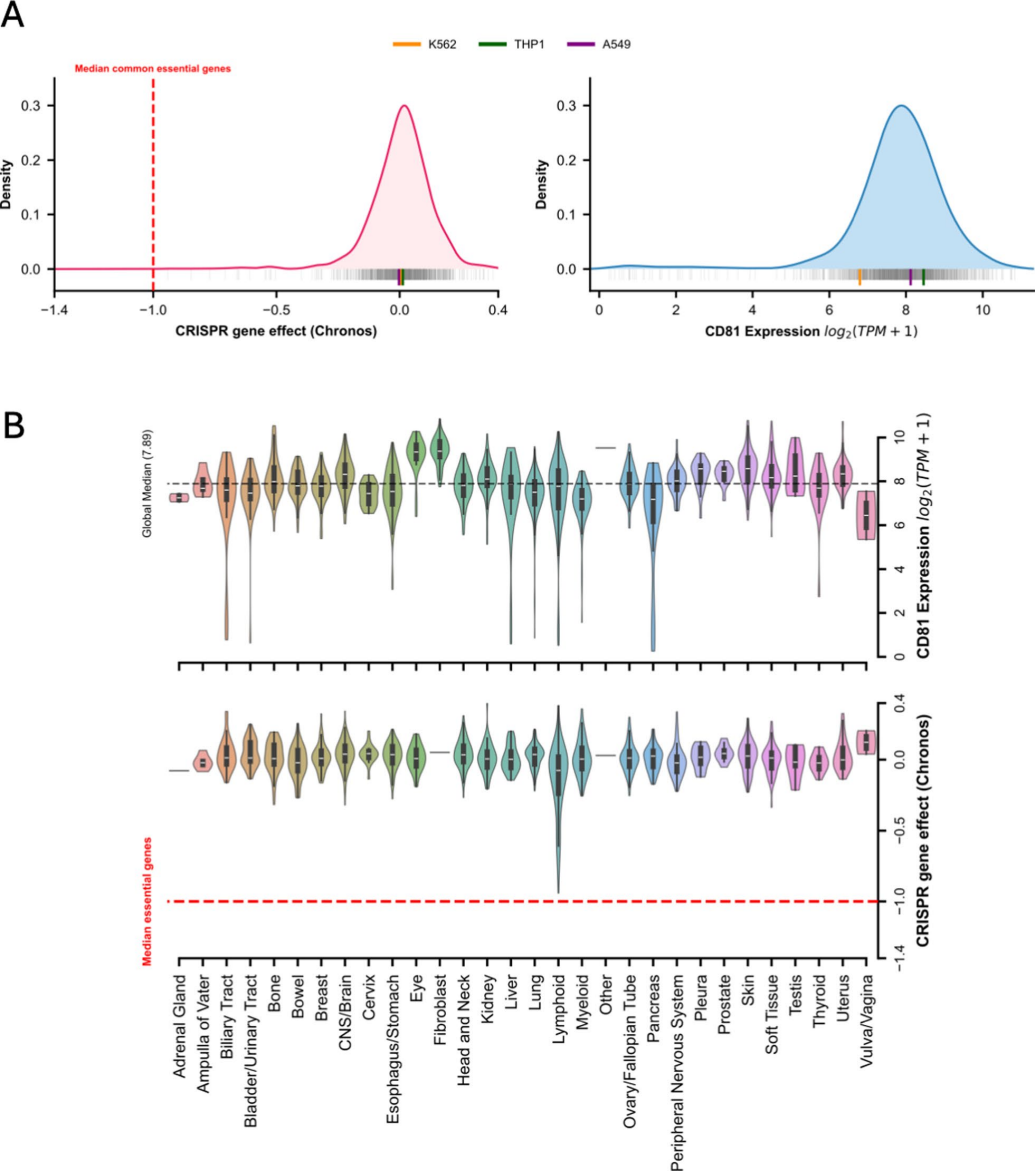
*CD81* is a nonessential gene that is highly expressed across the DepMap collection of cell lines. **A** Left: Density plot showing the distribution of *CD81* CRISPR disruption gene effects in 1187 cell lines (vertical grey bars); The red dashed line represents the median of common essential genes. Values near 0 indicate a low impact on cell viability. Right: Distribution of *CD81* gene expression across the same collection of cell lines, values are in log2 (TPM + 1). Three cell lines used in this study are highlighted. **B** Top: Violin plots showing *CD81* expression (log2 (TPM + 1)) across cell lines from 30 lineages. The horizontal dashed line indicates global median expression across the collection. Bottom: Distribution of *CD81* CRISPR gene effect across the same lineages. The red dashed line represents the median threshold for essential genes, showing a consistent non-essential distribution for *CD81*

To evaluate dCas9 functionality across different cell types, we selected a single guide RNA (sgRNA) targeting the promoter of the human *CD81* gene, encoding a non-essential and ubiquitously expressed membrane protein. To confirm the non-essentiality and ubiquitous expression of *CD81*, we analysed CRISPR screen and RNA-seq data from a collection of cell lines available on DepMap [12]. *CD81* was found to be non-essential in any of the tested cell lines (log2 gene effect less than − 1; Fig. 1A, left panel and 1B, bottom panel). However, 14 out of 1186 (1,1%) tested cell lines have a log2 gene effect less than − 0.5 (Supplemental Table S1). *CD81* is expressed in the majority of cell lines (Fig. 1A, right panel and 1B, top panel; median expression = 64,4 TPM), while only 18 (1%) have an expression lower than 1 TPM (Supplemental Table S1). Therefore, *CD81* is non-essential and highly expressed in a majority of cell lines, providing a reliable readout for CRISPRi experiments in a large panel of cell types. However, it is important to verify the essentiality and expression of *CD81* for any new cell line.

**Fig. 2:**
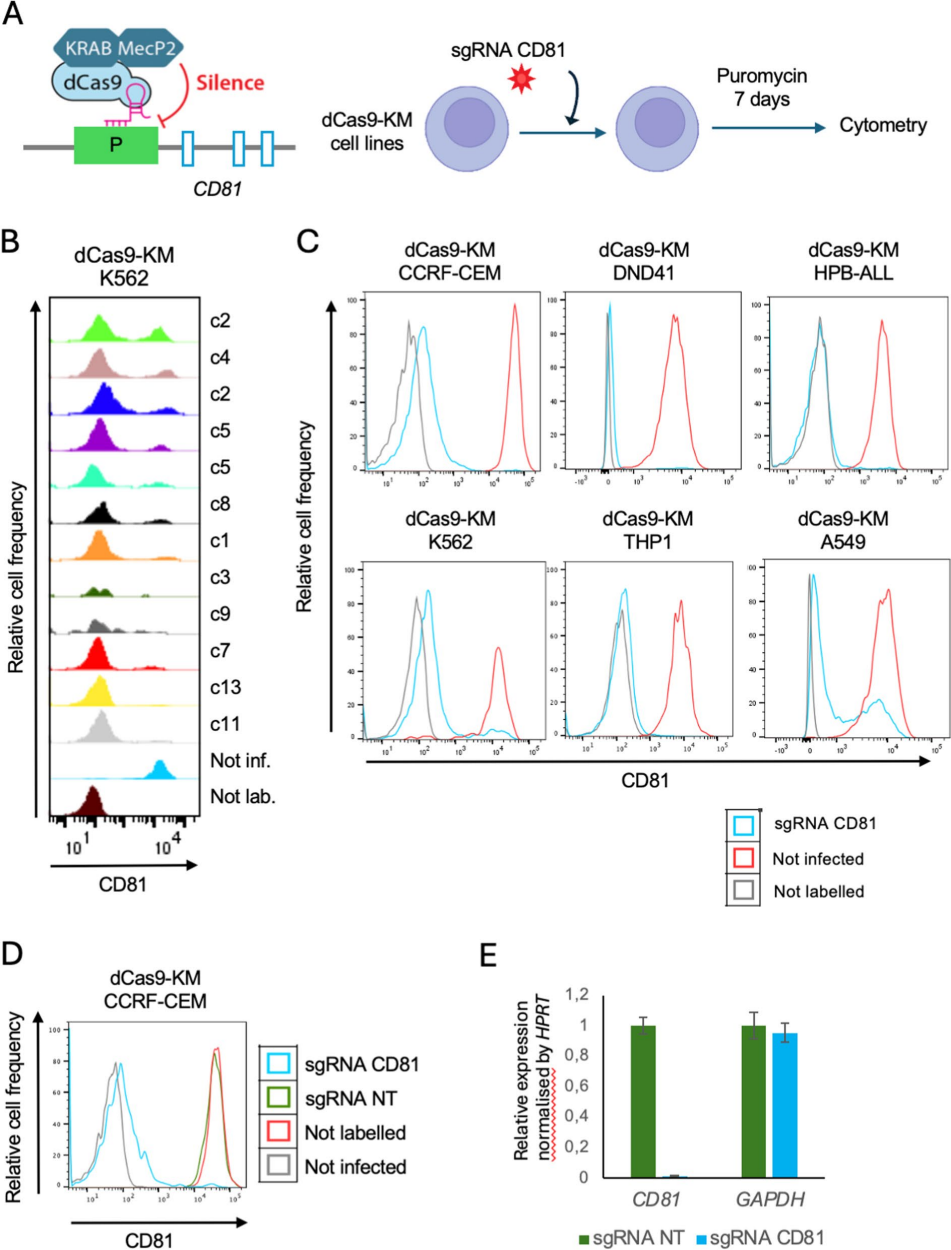
Assessment of dCas9-mediated repression using sgRNA-CD81. **A** Schematic overview of the sgRNA-CD81 transduction workflow. **B** Flow cytometry analysis of CD81 surface expression for several dCas9-KM K562 clones transduced with the sgRNA-CD81 vector. **C** Flow cytometry analysis of CD81 surface expression for representative clonal cells derived from the indicated cell lines. **D** Flow cytometry analysis of CD81 surface expression in CCRF-CEM dCas9-KM clonal cells transduced with the sgRNA-CD81 or sgRNA-NT vectors. **E** RT-qPCR analysis of *CD81* and *GAPDH* expression in CCRF-CEM dCas9-KM clonal cells transduced with the sgRNA-CD81 or sgRNA-NT vectors. The expression is normalised relative to HPRT gene expression.

To evaluate CRISPRi efficiency, we transduced different cell lines with a lentiviral vector expressing the dCas9-KM cassette. These included T-acute lymphoblastic leukemia (T-ALL; CCRF-CEM, DND-41 and HPB-ALL), chronic myelogenous leukemia (CML; K562), acute monocytic leukemia (AML; THP-1), and non–small cell lung cancer (NSCLC; A549) cell lines. After clonal selection, clones were infected with CD81-sgRNA, and CD81 surface expression was measured. As illustrated in Fig. 2A, the workflow involved sequential steps of stable dCas9-KM integration, single-cell cloning, and subsequent sgRNA-CD81 transduction.

To isolate dCas9-KM cells with high CRISPRi efficiency, we assessed CD81 repression after limited cell dilutions. Flow cytometry analysis revealed variability among individual clones, with some clones displaying stronger CD81 knockdown than others. Figure 2B shows representative K562 clones, where some clones exhibited near-complete CD81 depletion (e.g., c11, c13 clones) while others showed partial reduction. This highlights the importance of clonal selection to identify cell lines with maximal and reproducible dCas9 activity before downstream CRISPRi experiments. A similar clonal selection was performed for all the aforementioned cell lines. As shown in Fig. 2C, selected clones from all tested cell lines consistently displayed strong CD81 repression after the sgRNA-CD81transduction, confirming the robustness of this workflow across multiple cellular types.

Use of the CRISPRi system might result in artifactual deregulation of gene expression. Therefore, to validate our approach, we assessed CD81 expression using a non-target sgRNA in a CCRF-CEM dCas9-KM clone (Fig. 2D). As expected, transduction of the non-target sgRNA did not impact CD81 surface expression. In addition, it might be limiting for some laboratories to assess CD81 surface expression by flow cytometry. Therefore, we implemented an RT-qPCR strategy to assess *CD81* gene expression in the same CCRF-CEM dCas9-KM clone. We observed a dramatic decrease in *CD81* mRNA levels after transduction with the sgRNA targeting the *CD81* promoter as compared with the non-target control, while a non-targeted gene (*GAPDH*) remained unaffected (Fig. 2E).

## Conclusions

We generated and functionally validated dCas9-expressing haematological and solid tumor cell lines. The most efficient clones were selected based on dCas9-KM activity using an sgRNA targeting the *CD81* promoter. As CD81 is a membrane protein, a simple flow cytometry staining assay allowed us to assess its expression, providing a reliable readout for dCas9 activity. However, it is also possible to assess *CD81* gene expression by RT-qPCR.

Furthermore, we highlight the critical importance of selecting cell clones displaying high dCas9-KM activity, as the success of this technique strongly depends on robust repression efficiency. To achieve this, we recommend generating single-cell clones after stable integration of the dCas9-KM cassette, followed by functional testing using the CD81 sgRNA. By measuring CD81 expression via flow cytometry, it becomes possible to assess dCas9 repression efficiency and select clones with the strongest gene silencing potential for downstream experiments. Although we have not observed loss of CRISPRi-mediated inactivation after growing the clonal cells for several months, it is possible to lose activity over time due to clonal heterogeneity or revertant clones (e.g., loss of the dCas9-KM cassette). Therefore, we advise systematically using the sgRNA-*CD81* to verify CRISPRi efficiency over-time.

## Materials and methods

### Analysis of essentiality and gene expression

CRISPR essentiality and expression of CD81 across 1187 cell lines were retrieved from DepMap (depmap.org; Public release 25Q3; Supplemental Table S1) [12]. Data was plotted using RStudio (2022.02.0 + 443), under version R (4.1.3).

### Cell culture

HEK293T cells were cultured in DMEM high glucose (Gibco #41,965-062) supplemented with 10% fetal bovine serum (FBS; Atlanta Biologicals), 1% penicillin–streptomycin, and 2 mM L-alanyl-L-glutamine (GlutaMAX, Gibco #25,030,081). The T-cell acute lymphoblastic leukemia (T-ALL) cell lines CCRF-CEM (ATCC CCL-119), HPB-ALL (DSMZ ACC-483), and DND-41 (DSMZ ACC-525), the myeloid cell line K562 (DSMZ ACC-10), and the monocytic cell line THP-1 (DSMZ ACC-16) were maintained in RPMI 1640 medium (Gibco #21,875) supplemented with 10% FBS. The A549 cell line expressing dCas9-KRAB-MeCP2 was kindly provided by Bernard Mari (Institut de Pharmacologie Moléculaire et Cellulaire, Nice) and cultured in DMEM high glucose (Sigma-Aldrich #D5648) supplemented with 10% FBS. All cell lines were cultured at 37 °C in a humidified atmosphere containing 5% CO₂.

### Vector cloning

The sgRNA sequence targeting the human *CD81* promoter (sgRNA-CD81: 5′-GCCTGGCAGGATGCGCGGTG-3′) was obtained from a protocol provided by the Genetic Perturbation Platform from the Broad Institute (https://portals.broadinstitute.org/gpp/public/dir/download%3Fdirpath%3Dprotocols/production%26filename%3DdCas9_Activity_Assay_CRISPRi_Sep2020.pdf&ved=2ahUKEwjupMXhtZeSAxUeU6QEHVZUKxAQFnoECB8QAQ&usg=AOvVaw10ENP7-u8BBGx83ifhD-m5). A non-target control was obtained from the negative control list of the hCRISPRi-v2 library [13] (sgRNA-NT: 5′-GGGAACGACTATGACCGCCA-3'). The sgRNAs were cloned into the CROPseq-Guide-Puro vector (Addgene, #86,708), following the protocol described by Datlinger et al. [14]. Briefly, 74-mer oligonucleotides synthesized by Eurofins Genomics were diluted to 100 µM and annealed. The CROPseq vector was digested with BsmBI (NEB), gel-purified using the PureLink Quick Gel Extraction Kit (Invitrogen), and assembled with the sgRNA using NEBuilder HiFi DNA Assembly Master Mix (NEB #E2621L). Competent *E. coli* (Lucigen Endura) were transformed, and plasmids were purified using the EndoFree Maxiprep Kit (Qiagen #12,362).

### Lentivirus production

Lentiviruses were produced in HEK293T cells using the calcium phosphate transfection method. Cells were co-transfected with the lentiviral expression vector, gag-pol packaging plasmid, and VSVG envelope plasmid (Addgene, #12,259). Supernatants were harvested at 48 and 72 h post-transfection, filtered through 0.45 μm filters (Merck-Millipore #SLHV033R), and concentrated using Lenti-X Concentrator (Takara Bio).

### Generation of dCas9-KRAB-MeCP2 cell lines

CCRF-CEM, DND41, HPB-ALL, K562 and THP-1 cells were transduced with lentivirus encoding dCas9-KM (Addgene #122,205) at a multiplicity of infection (MOI) of 10. Transductions were performed twice, four days apart. Three days after the second infection, cells were selected with 10 µg/mL blasticidin (Thermo Fisher) for 14 days. Single-cell cloning was performed by limiting dilution at 0.5 cells per well in 96-well plates. Clones were expanded for two weeks before downstream experiments.

### sgRNA transduction

Cell lines were transduced with the sgRNA-CD81 or sgRNA-NT CROPseq lentivirus. Three days post-transduction, puromycin (2.5 µg/mL) was added for seven days.

### Flow cytometry

CD81 knockdown was assessed by flow cytometry. Cells (100,000) were stained using APC-conjugated anti-human CD81 antibody (BioLegend #349,510, clone 5A6) at 1:100 dilution and analyzed using a BD LSRFortessa X-20 cytometer equipped with UV, violet, blue, yellow, and red lasers. Data analysis was performed using FlowJo software. Fluorescence minus one (FMO) control was used to define gating strategies.

### Gene expression analyses

Total RNA was extracted using the RNeasy Plus Mini Kit (Qiagen) following the manufacturer’s protocol. One microgram of RNA was reverse-transcribed with SuperScript™ VILO™ Master Mix (Thermo Fisher Scientific, #11,755,250). RT–qPCR was performed using SYBR Green Master Mix (Thermo Fisher Scientific) on a QuantStudio™ 6 Flex (Thermo Fisher Scientific) instrument, with 1:10 diluted cDNA. Relative expression was analysed by the 2^ΔΔCT method, normalised to HPRT expression. Each group was tested in three independent RNA and cDNA preparations, and the mean ± standard deviation was calculated relative to the no-target control. Primers: CD81 F 5(TTCTCCGGGAAGCTGTACCT), CD81 R (ACCATGCTCAGGATCATCTCG), HPRT F (GGGTGTTTATTCCTCATGGAC), HPRT R (CTCCCATCTCCTTCATCACA), GAPDH F (CCCACTCCTCCACCTTTGAC) and GAPDH R (CCACCACCCTGTTGCTGTAG).

## Supplementary Information

Additional file 1.

## Data Availability

All data generated or analyzed during this study are included in this published article.

## Abbreviations

sgRNA: Single guide Ribonucleic Acid
TPM: Transcripts *per* Million
NT: Non-target
RT–qPCR: Reverse Transcription quantitative Polymerase Chain Reaction
KRAB: Krüppel-Associated Box
MeCP2: Methyl-CpG-Binding Protein 2
CRISPR: Clustered Regularly Interspaced Short Palindromic Repeats
CRISPRi: CRISPR interference
Cas9: CRISPR-associated protein 9
dCas9: Dead Cas9
dCas9-KM: DCas9-KRAB-MeCP2
CD81: Cluster of Differentiation 81
RPMI: Roswell Park Memorial Institute
DMEM: (Dulbecco’s Modified Eagle Medium
FBS: Fetal bovine serum
µg: Microliter
mL: Millilitre
FMO: Fluorescence minus one
T-ALL: T-acute lymphoblastic leukemia
CML: Chronic myelogenous leukemia
AML: Acute monocytic leukemia
NSCLC: Non–small cell lung cancer
